# Composite global indicators from survey data: the Global Economic Barometers

**DOI:** 10.1007/s10290-021-00449-8

**Published:** 2022-02-04

**Authors:** Klaus Abberger, Michael Graff, Oliver Müller, Jan-Egbert Sturm

**Affiliations:** 1 KOF Swiss Economic Institute, ETH Zürich, Zürich, Switzerland; 2 CESifo, Munich, Germany

**Keywords:** Business cycles, Composite indicators, Covid-19 pandemic, Leading indicators, Coincident indicators, Partial least squares, Real-time simulations, World economy, E32, E37

## Abstract

This paper presents a coincident and a leading composite monthly indicator for the world business cycle—the Global Economic Barometers. Both target the world’s output growth rate and consist of economic tendency surveys results from many countries around the world. The calculation of these indicators comprises two main stages. The first consists of a variable selection procedure, in which a pre-set correlation threshold and the targeted leads to the reference series are used as selection criteria. In the second stage, the selected variables are combined and transformed into the respective composite indicators, computed as the first partial least squares factor with the reference series as response variable. We analyse the characteristics of the two new indicators in a pseudo real-time setting and demonstrate that both are useful additions to the small number of indicators for the global business cycle published so far. Finally, yet importantly, the Barometers were quick to plunge in the beginning of March 2020 and have since then given a reliable real-time reflection of the economic consequences of the Covid-19 pandemic.

## Introduction

In recent years, the amount of information from economic tendency surveys, which is potentially useful for explaining global economic developments, has increased significantly.^1^ New surveys amongst firms, consumer and experts have been launched and existing surveys have been expanded by including additional industries and sectors of countries across the globe. Furthermore, a number of previously quarterly surveys are now conducted at monthly frequency. Many of these surveys now have a history that is long enough to be considered in the construction of new composite indicators. At the same time, the ever-stronger interconnectedness of consumers, firms and governments across the world has considerably increased the interest in timely and transparent information on the state and the development of the global economy.

On this basis, we have constructed two composite indicators for the world economy—the Global Economic Barometers. One is designed as a coincident indicator, the other as leading the world economy by about half a year.^2^ Referring to a broad set of underlying variables aggregated by partial least squares with the world business cycle as response variable, the Barometers extract strong signals from a large global information set, where much of the idiosyncratic noise is cancelled out. We can hence avoid low-pass filtering of both input and output series and the well-known problems associated with it, such as endpoint instability and phase shifts. Another important feature of the Barometers is that contrary to most other composite indicators, where the underlying set of variables rarely changes, the Global Barometers automatically adapt to changing circumstances and relationships, allowing new variables to enter and others to be dismissed; the composition as well as the estimated parameters of the Barometers are adjusted for every release. Our real-time analyses confirm that the new Barometers are indeed often either faster in detecting or better in capturing cyclical movements of the world economy than the few other indicators at the world level that are presently available. Last but not least the Barometers have delivered a reliable real-time reflection of the economic consequences of the Covid-19 pandemic.

The rest of this paper is set up as follows. After giving an overview of composite indicators at the world level in Sect. 2, the construction of the Global Economic Barometers is explained in Sect. 3. The Barometers, their properties and time series characteristics along with summaries of several robustness checks are presented in Sect. 4. We then compare the Barometers to other major composite indicators for the world economy (Sect. 5). Section 6 looks at how the Barometers picked up the initial Covid-19 pandemic shock and the subsequent infection waves that affected the global economy to various degrees. Section 7 concludes.

## Composite indicators for the world economy

The concept of a global business cycle, and evidence for it, has been discussed in the academic literature for a long time.^3^ However, it is the impact of the 2007 financial crisis and the resulting 2008/9 Great Recession as well as the subsequent events, from the euro crises to the US move towards protectionism and the ongoing pandemic originating from China’s Wuhan in the end of 2019, which have now brought the close interconnectedness of the world’s economies to the public’s attention. Recent discussions about trade barriers and the Covid-19 pandemic have also raised questions about their impact on the global economy. Indicators for the global economic situation and outlook are hence clearly of interest. However, in contrast to economic indicators at the country level, there are very few regularly published indicators at the world level. The existing ones can be divided into four broad categories. First, indicators based on quantitative (official) statistics. Second, indicators based on price or volume data. Third, indicators based on surveys of experts, companies, or consumers. Fourth, composite indicators based on different information, some of which being developed at a more ad hoc basis in academic research papers. We shall turn to these now.

### Indicators based on quantitative country statistics

Various institutions calculate international aggregates from quantitative data for the economic situation at the country level, such as industrial production or export statistics. The United Nations (UN), for example, publishes a global industrial production time series.^4^ The CPB Netherlands Bureau for Economic Policy Analysis calculates a world trade volume indicator and an industrial production indicator.^5^ The OECD publishes industrial production for its member countries and for the OECD as an aggregate as well as various sub-aggregates,^6^ but not for the world as a whole.

Although these international indicators are based on data that are published earlier than GDP, their usefulness is impaired by considerable publication lags.

### Price and volume measures

Prices or quantities of goods or services that are measured at the global level are sometimes directly used as indicators for global economic developments. Examples are commodity prices or freight rates. In addition, volume measures of commodity production or freight volume can reflect useful information, although one would expect that prices react often before volumes. Examples for commodity price indicators are the HWWI commodity price indices^7^ or the Baltic Dry Index,^8^ which covers specific freight rates. Examples of volume indicators are the global steel production published by the World Steel Association.^9^ Another example is freight transported by plane, published by the IATA.^10^

A potential problem with indicators of this category is that they could be driven not only by the world business cycle but also by structural changes that do not directly link to current economic conditions. For example, prices are influenced when new ship fleets are put into operation, which may be a long time after the demand for increased shipping capacity signalled increases in expected trade.

### Surveys (experts, firms, consumers)

Indicators for country-specific business cycles often resort to economic tendency surveys. These surveys are conducted among either firms, consumers, or experts. There are only a few surveys with an international scope. An expert survey of this type, which has recently been discontinued, was the ifo World Economic Survey.^11^ Markit Economics publishes a global PMI indicator using results of their national business surveys.^12^ A global consumer confidence indicator is published by The Conference Board.^13^ Other institutions collect national survey data and calculate supra-national results. For example, the OECD publishes a business confidence indicator^14^ and a consumer confidence indicator^15^ for the OECD aggregate in its report “OECD Main Economic Indicators”. In addition, the European Commission calculates various survey-based indicators for the EU, reflecting the situation in Europe.^16^

### Composite indicators

A few institutions calculate composite indicators by broadening the scope beyond national economic tendency survey results and publish these regularly. The OECD maintains a system of composite leading indicators for its member countries and six important non-member countries.^17^ It publishes composite indicators for various regions, including an indicator for the OECD member states (called OECD total) and an indicator that covers the OECD member states plus the six non-member economies (OECD total + Major six NME). The Conference Board has a broader scope; it calculates a leading and a coincident indicator for the global economic cycle.^18^ Both institutions construct their composite indicators based on expert knowledge to determine the inclusion of potential variables.

Furthermore, some of such indicators are presented and discussed in academic research papers, but not published on a regular basis, presumably due to lack of institutional support or perceived interest. This notwithstanding, the findings provide valuable guidance for developing new indicators like ours. An indicator that fits into this category is the global GDP nowcast based on a single input series—the global Markit PMI—by Rossiter (2010). Stratford (2013) shows in a real time simulation that resorting to a regression-based combination of global indicators on trade, prices and sentiment became helpful in forecasting world GDP since the global recession of 2008/9, but not in the years before. Drechsel et al. (2014) resort to a handful of indicator variables reflecting regional aggregates for short-term forecasts of quarterly world GDP growth and find that they outperform early releases of the corresponding IMF forecasts. Golinelli and Parigi (2014) present a monthly bridge equation model for nowcasts and short-term forecasts of the GDP of ten economies that jointly contribute some 70% of world GDP based on a mix of “hard” indicators, prices, and survey data. Importantly, their real-time simulations show that predictive accuracy is markedly overstated when referring to the last vintages of indicator and target variables. Camacho and Martinez-Martin (2015) construct a Markov-switching factor model for the global business cycle from six hand-selected variables and apply it to forecast NBER type recessions. Camba-Mendez et al. (2015) present composite indicators targeting industrial production in the euro area. They alternatively resort to a hand-selected set of indicators as well as to a large data set from which subsets are generated by automatic statistical procedures. Cuba-Borda et al. (2018) estimate a Global Conditions Index based on world industrial production, world retail sales, the global PMI and world quarterly GDP growth. They show that a real-time forecast of their index based on the latest information set is closely related to GDP growth and helpful to quantify recession probabilities.

Although we may have overlooked some pieces of research in this field, the number of publications is still quite limited. Yet, the issue has already been approached with a wide variety of methods and underlying data. To the best of our knowledge, our Barometers are a new approach that combines some of the most promising features to produce particularly timely and at the same time robust signals of short-term developments of the global economy. Rather than relying on a specific type of tendency survey at a time, they simultaneously refer to tendency surveys amongst firms, consumer, and experts. In addition, the scope extends beyond the OECD; information is taken from more than 50 countries all over the world. Closest to our approach in technical terms is one of the alternatives of Camba-Mendez et al. (2015), where subsets are generated from a large pool of variables by automatic statistical procedures, but their indicator targets industrial production in the euro area.

## Construction of the Global Economic Barometers

The Global Economic Barometers differ from the limited number of previous indicators by combining the following seven features. They (1) target the global economic situation quantified by the world GDP growth, (2) deliver a coincident and a leading indicator, (3) resort to data from all over the world, which ensures a truly global perspective, (4) draw exclusively on economic surveys, which exhibit very short publication lags (usually only a number of days) and practically no or only minor revisions, (5) are published at a monthly frequency, (6) are automatically adjusted to changing patterns of correlations and leads/lags, allowing for changing variable compositions and aggregation parameters with each monthly release and (7) refer to seasonally adjusted input series but do without low-pass filtering at all stages, which would make the Barometers look smoother but leads to phase shifts and/or revisions at the right margins, i.e. where the Barometers matter most to provide timely information.

### Quantifying the global business cycle: the reference series

The construction of our Barometers starts with the computation of a quantitative measure for the global business cycle to be used as reference series in the variable selection and aggregation procedures. Business cycles reflect the common information and synchronicity simultaneously observed in different branches and demand components. In practice, macroeconomic analyses of business cycles often refer to fluctuations of GDP, as the latter is the aggregate of value added within a specified period, so that it can be expected to reflect major co-movements of economic activity. We follow this practice and thereby focus on growth rates. Expansions (contractions) are thus identified by increasing (decreasing) growth rates.

For the world economy, the International Monetary Fund (IMF) publishes an estimate of the year-on-year (y-o-y) real quarterly GDP growth rate at quarterly frequency. To this end, the IMF aggregates national GDPs to global GDP at purchasing power parity (PPP). The last vintage of the IMF GDP series available when finalising this paper in August 2021 is dated 2018Q4, as the IMF has presently suspended the publication of this time series.^19^

Our variable selection procedure is based on cross-correlations of the potential indicator variables on the one hand, which are mostly monthly, and the reference series on the other. We must hence either aggregate all monthly variables into quarterly frequency or disaggregate the quarterly global GDP series into monthly frequency. We choose the latter, because it is informationally more efficient and allows for a more fine-grained analysis of the lead/lag-relationship between the variables and the reference series. In addition, as the latest Covid-19 related recession demonstrated once more, for assessments of the economic situation, monthly time series can be much more informative than those at quarterly frequency.

Accordingly, we first compute real quarterly global GDP levels from the IMF’s real quarterly global GDP year-on-year growth rate. We then submit the quarterly GDP level series to seasonally adjustment and disaggregate it with the Denton additive method. This method assures that the disaggregated monthly values add up to the original quarterly value.^20^ From the resulting monthly GDP series, we can compute growth rates at a monthly frequency, either month-on-month (m-o-m) or quarter-on-quarter (q-o-q) or year-on-year (y-o-y). An obvious disadvantage of using m-o-m or q-o-q growth rates is their high volatility. Since our Barometers are designed to signal the underlying business cycle, and not high frequency fluctuations triggered by distortions or seasonality, the comparatively smooth y-o-y growth rate of monthly GDP will serve as our reference series.

**Fig. 1:**
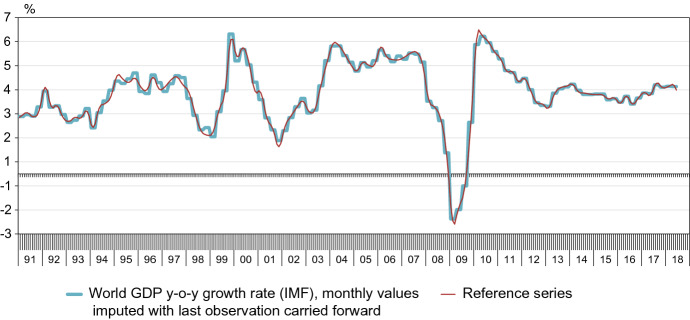
IMF series and reference series

Figure 1 plots the resulting reference series with the original quarterly year-on-year growth rate as published by the IMF. (For illustrative purposes, the monthly values are imputed with the last observation carried forward for three months.)

### The pool of variables

The Global Barometers are designed as survey based composite indicators, i.e. we only consider variables from consumer, business, and expert tendency surveys. In total, we have assembled a set of 1681 variables from all over the world, for which we a priori expect that they have a close relationship with the world business cycle.^21^ Table 1 shows the number of variables available broken down by major regions. Table 2 shows the variable count by sector and Table 3 by indicator type.

**Table 1:** Variable count by region

Region	Variable count	Percentage of total
Europe	995	59%
Asia and Pacific and Africa	359	21%
Western Hemisphere	327	19%

**Table 2:** Variable count by sector

Sector	Variable count	Percentage of total
Economy	405	24%
Retail and wholesale trade	271	16%
Construction	205	12%
Industry	657	39%
Services	143	9%

**Table 3:** Variable count by indicator type

Indicator type	Variable count	Percentage of total
Expectations	527	31%
Confidence	283	17%
Present Situation	871	52%

### The variable selection procedure

The variable selection procedure is designed to identify indicator time series with a sufficiently high and economically meaningful correlation with the reference series, imposing either coincidence with the reference series or a targeted lead of about six months. This approach is related to variable selection methods based on regressions and “hard thresholding” commonly used in factor models. In the context of macroeconomic time series forecasting, Bai and Ng (2008) have suggested a “targeted predictors” approach, where variables enter a principal components extraction conditional on that they are significantly correlated with a pre-determined target variable. This approach has since then been repeatedly applied to pre-select indicator variables reflecting national business cycles before extracting their common factor.^22^ Hard thresholding based on regression coefficients is a common approach in partial least squares regression as well.^23^ In contrast to the implementations referred to above, however, our approach in addition evaluates lead/lag-relationships of potential indicator variables with the reference series.

Before submitting the variables to the selection procedure, we determine the expected sign of the association between the variables on the one and the reference series on the other hand.^24^ In the automatic variable selection procedure, only variables whose correlations with the reference series have the expected sign are considered, so that spurious correlations should largely be ruled out.

To remove seasonality, which is common in business cycle related data, we send all indicator variables through the X-13-ARIMA-SEATS seasonal adjustment procedure developed by the United States Census Bureau. Practically all series display a smaller or larger layer of noise, but we deliberately do not apply low pass filters, as such attempts to increase the signal-to-noise ratio are bound either to lead to instability at the right margins of the series or to induce a phase shift. As far as the noise in the selected indicator variables is uncorrelated, it will cancel out when the variables are aggregated.

Depending on the variables’ characteristics, we add various transformations to the pool. The potential transformations are the logarithm and/or differences (1/3/12-month differences for monthly variables; 1/4-quarter differences for quarterly variables). For variables that are likely to contain a unit root, we add the time series’ differences to the variable pool and remove the original level variable. For stationary variables, we allow both levels and differences to enter. Since the transformations are either differences or monotone (logarithm), we assign the same expected sign to the transformations as to the original variables.

Prior to initializing the variable selection procedure, we deal with the so-called “ragged-edge” problem, which occurs when series display missing observations at the end of the sample due to publication lags or temporary suspensions of publication. To secure a balanced panel at the end of the sample, we shift the affected variables forward by the publication lag. This corresponds to what Altissimo et al. (2007) call “vertical realignment”. In comparison to other methods devised for dealing with the ragged-edge problem, like filling them with forecasts based on autoregressive models, our method does not lead to revisions in the past when a new vintage is released—something we want to avoid as far as possible. The determination of the expected publication lags is based on experience. Specifically, we take the second-largest publication lag a variable has experienced in the preceding 12 months. When actual publications lags happen to be longer than this, the missing observations at the end of the panel are imputed with the expectation–maximization (EM) algorithm for partial least squares.

For each variable, we compute the cross-correlation with the reference series spanning the range from a lead of 12 months to a lag of 12 months. The lead of a variable to the reference series is then defined as the lead where the absolute cross-correlation is maximized. The sample period for computing the cross-correlations is set to a 10-years rolling window. This reduces the dependence on distant past observations and allows for timely adjustments of the variable selection. The end of the 10-years window is set to the month of the last observation of the reference series. In practice, the 10-years window shifts forward by three months every quarter, i.e. when our reference series is updated with an additional quarterly data point.^25^

We determine a coincident and a leading indicator for the world business cycle, where the latter is intended to lead the reference series by about six months. To incorporate this in the algorithm, to be selected, a variable must meet the following criteria:

- The variable must have valid observations throughout the 10-years sample period.
- The recorded lead of the variable must fall within a 7-month window centred at the desired lead of either 0 or 6 months. Hence, the lead must fall between − 3 and + 3 months for the coincident indicator and between + 3 and + 9 months for the leading indicator.
- The sign of the cross-correlation at the recorded lead must comply with the exogenously imposed sign restriction.
- The cross-correlation at the recorded lead must pass the 5% significance threshold.

To compute the significance level, we use the fact that the sample cross-correlations between two independent stationary time series $x$ and $y$ are asymptotically normally distributed with

1 $$\hat{r}_{xy} \sim AN\left( {0,T^{ - 1} \left[ {1 + 2\mathop \sum \limits_{j = 1}^{\infty } r_{x} \left( i \right)r_{y} \left( j \right)} \right]} \right),$$

where $r_{x} \left( i \right)$ and $r_{y} \left( j \right)$ are the autocorrelations of $x$ and $y$ at lag $j$ and $T$ denotes the sample size (Brockwell & Davies, 1987, p. 400). The maximum order of the autocorrelation in Eq. (1) is chosen according to the Schwert criterion $\left\lfloor {4(T/100)^{1/4} } \right\rfloor$ (Schwert, 1989, p. 151). To ensure that the finite-sample estimate of the variance is positive, we use the Bartlett kernel as in Newey and West, (1987). The usual z-test statistic is computed in the form of the ratio of the observed cross-correlation and its standard deviation. We perform a one-sided test for variables with pre-defined sign restrictions and a two-sided test for variables without.

The selection criteria are applied to all transformations of the same original variables. When multiple transformations meet the above criteria, only the one with the maximum correlation at the desired lead is retained. Both subsets of selected variables are then aggregated by extracting the first partial least squares factors. To ensure an adequate representation of the major global regions, regional factors are extracted from regional subsets of the selected variables and then aggregated to the Global Economic Barometers with regional GDP shares.

### Univariate partial least squares

Consider the multilinear regression

$$y = X\beta + \varepsilon$$

with a $T \times 1$ response variable $y$ and a $T \times N$ matrix $X = \left[ {x_{1} ,x_{2} ,...,x_{N} } \right]$ of $N$ explanatory variables. The ordinary least squares (OLS) solution is given by

$$\hat{\beta } = (X^{\prime } X)^{ - 1} X^{\prime } y.$$

If $N > T$, the matrix $X^{\prime } X$ is singular and no unique solution for $\hat{\beta }$ exists. When $N \le T$, but $N$ is large relative to $T$, OLS is prone to overfitting, such that the model fits the in-sample data including their noise well but fails to give good out-of-sample predictions. Partial Least Squares (PLS) regression circumvents these problems by constructing a smaller number of new explanatory variables, often called latent variables or *factors*, that explain as much of the covariance between $X$ and $y$ as possible. The PLS factors are linear combinations of the variables in $X$ and uncorrelated with each other. Each factor ${f}_{k}$ has a corresponding weight vector${w}_{k}$, such that${f}_{k}=X{w}_{k}$. PLS solves the following maximization problem to find the first PLS factor:

2 $${\text{max}}_{{w_{k} }} cov\left( {Xw_{k} ,y} \right)\;{\text{subject}}\;{\text{ to}}\;\left| {\left| {w_{k} } \right|} \right| = 1$$

Hence, the weights $w_{k}$ are chosen such that the covariance between the factor $f_{k} = Xw_{k}$ and $y$ is maximized. For subsequent factors the orthogonality constraint ${f}_{k}^{^{\prime}}{f}_{i}=0, \forall i<k$ is added to the above maximization problem to make sure the factors are uncorrelated with each other.

In our application, *X* consists of the variables selected by the selection procedure; and the response variable $y$ is given by the reference series. In line with one of the variable selection criteria, the in-sample period is set to a 10-years window that ends in the month of the last observation of the reference series.

We then extract two first PLS factors *f*_1_, one from the pre-selected subset of coincident variables and the other from the pre-selected leading subset. Finally, to ease communication, the two resulting standardized variables are scaled to means of 100 and standard deviations of 10 across their in-sample periods. The resulting time series are released as the coincident and the leading Global Barometers.

Thus, we refer to PLS for dimension reduction, a method that has been found useful in applications with many independent variables and in in the presence of collinearity. Compared to the widely used principal component method (see next paragraph) PLS is a “supervised” method in the sense that it makes use of the response $y$ to find factors that are related to it. As James et al., (2013, p. 237) put it: “Roughly speaking, the PLS approach attempts to find directions that help to explain both, the response and the predictors. In contrast, principal components is in that sense unsupervised because it only uses $X$ to identify factors.”

### Partial least squares versus alternative statistical aggregation procedures

In PLS regressions, both $y$ and $X$ inform the construction of the factors. In contrast, principal components (PC) regressions choose the factors such that they explain as much of the variance in $X$ as possible. Instead of Eq. (2), PC regressions solve the following maximization problem to find the factors:

$${\text{max}}_{{w_{k} }} {\text{var}} \left( {Xw_{k} } \right)\quad{\text{subject }}\;{\text{to}}\quad\left| {\left| {w_{k} } \right|} \right| = 1\text{.}$$

Amongst others, Helland (2001) and Yoon et al. (2015) argue that PLS regressions are more adequate to combine large numbers of variables ($X$) into a latent variable that is supposed to correlate with another series ($y$). Particularly important is that whilst PC extraction maximizes the covariance of $X$, PLS maximizes the covariance between $y$ and $X$, which in our case amounts to a direct alignment of the first factor to the reference series. With PC, it is left to an informed variable selection procedure alone to make sure that the first component is related to $y$.

Moreover, in PLS regressions with standardized variables, the factor weights of the variables are proportional to their correlations with the reference series. Formally, for two variables ${x}_{1}$ and ${x}_{2}$ and their first PLS factor weights ${w}_{11}$ and ${w}_{12}$, the following holds: ${w}_{11}/{w}_{12}=corr({x}_{1},y)/corr({x}_{2},y)$. This allows for a straightforward interpretation of the factor weights relative to each other. But more importantly, the sign of a PLS factor weights is equivalent to the sign of the corresponding variables’ correlations with the reference series, i.e. ${sign(w}_{1i})=sign(corr\left({x}_{i},y\right))$. Remember that in the variable selection procedure, we only consider variables whose correlations with the reference series have a sign equivalent to the one expected. Because the sign of the PLS factor weights corresponds to that of the correlations with the reference series, the contributions of the selected variables to the PLS factor will always be of the correct sign and hence thematically correct. To give an example: A variable that indicates the business situation is supposed to contribute positively to the change of the Barometers if it increases and negatively if it decreases. We only consider such a variable if it has a positive correlation with the reference series and this in turn means it will have a positive PLS factor weight and consequently its contribution to the change of the Barometers will be positive if it increases and negative if it decreases. This is not guaranteed with PC regression. Even though a sign mismatch between the correlation and the PC factor weight and thus a contribution opposite to the one expected is a relatively rare occurrence, it represents a spurious relationship we want to avoid. Also, to interpret an indicator’s impact on the outcome of a PC regression, one must look at the signs of the variables’ factor loadings along with the corresponding factors’ correlations with the target series, which may amount to a tricky exercise, especially if large numbers of variables are involved, as repeated computations may exhibit unpredictable changes to the sign structure. Whilst the task of tracking joint signs is *in principle* manageable, the predictability of the correspondence of sign and impact in PLS greatly eases the task to interpret and communicate monthly outcomes, which is particularly useful for our purposes. Furthermore, with PLS, the relative weight of individual variables (i.e. ${w}_{11}/{w}_{12}$) is independent of the other variables included in the extraction. PC, on the contrary, increases the weight of variables according to their covariance with the other variables in the factor extraction independently from their association with a target variable. Common noise or seasonality across variables will thus increase their weights, which is not desirable.

Given our objective to compute a one-dimensional latent variable that weighs the variables in the extraction according to their congruence with the reference series and exhibits a loading structure that can be interpreted without considering an interdependent sign distribution, the above arguments clearly favour PLS over PC in our situation.

Another procedure related to PLS that can be used to analyse multidimensional variable sets ($X$ and $Y$) is canonical correlation analysis (CCA). Like PLS, CCA seeks a number of linearly independent factors for each set of variables. Unlike PLS, it is not the covariance but the correlation between the factors of the two sets that is maximized. If at least one of the variable sets is one-dimensional, CCA reduces to least squares regression (e.g. Bie et al., 2005). Since for our task $Y$ consists of the reference series and is one-dimensional, we would in practice be doing multilinear regression as described at the start of this section. This is not an option due to the large number of variables relative to the number of data points. The regularized version of CCA (RCCA) avoids the overfitting problem and is equivalent to Ridge regression in the context of a one-dimensional target variable. We do not explore RCCA in this paper, however, it is worth noting that the first PLS factor corresponds to the maximally regularized first RCCA factor (i.e. with the regularization parameter going to infinity, see Bie et al., 2005).

Yet another procedure that is frequently referred to for multivariate time series analyses is cointegration analysis. However, our approach is based on cross-sectional correlations between the reference series and indicator variables and aggregation by PLS. The time dimension is hence absent in our framework, at least in statistical terms, so that the cointegration concept does not apply here.

### Missing observations

Our variable sets include time series with quarterly frequency, where two out of three months are missing. In addition, time series with unexpected publication delays will have missing observations at the end of the series. To construct the PLS factor in the presence of missing observations, we use the expectation–maximization (EM) algorithm for partial least squares as proposed by Rännar et al. (1995), also often called iterative algorithm (e.g. Walczak & Massart, 2001).

As the world GDP growth rate underlying the reference series has a publication lag of up to seven months and since it is updated once per quarter, the response variable will inevitably lack observations at the end of the panel. We do not estimate missing observations in the reference series. Instead, we set the end of the 10-years in-sample period to the month of the last observation of the reference series. Out-of-sample, we use the in-sample PLS weights and loadings to compute the PLS factor.

### Regional aggregation

**Fig. 2:**
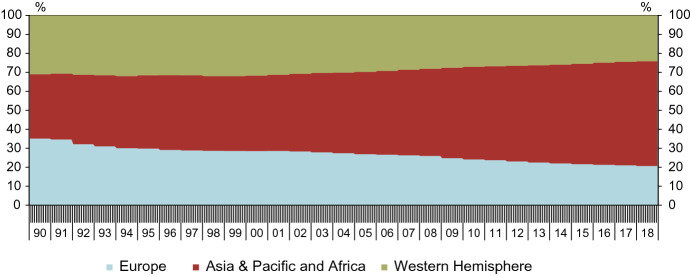
Regional shares of global GDP

As shown in Table 1 above, indicators from Europe make up close to 60 per cent of the variables in our pool. In contrast, Europe’s share of global GDP is much lower. Figure 2 plots the regional shares of global GDP over time, based on the 2019 vintages of the regional GDPs at purchasing power parity (PPP) as published annually by the IMF. In 2018, Europe’s share of global GDP was 21 per cent.

Over- or underrepresentation of particular regions may lead to an over- or underrepresentation in the number of selected variables and ultimately in their weight in the aggregation to the Barometers.^26^ Although PLS selects the weights of the variables via their relationship to world GDP, regional shocks, for example, could potentially distort the signal if the number of regional variables differs too much from the weight of the region for the world economy. As the Barometers are supposed to indicate the global business cycle, one option to deal with over- or underrepresentation of particular regions would be to compute regional indicators based on regional reference series and aggregate them with regional GDP weights to arrive at the Barometers. Unfortunately, regional GDPs are only available at yearly frequency (and not quarterly, as the global aggregate). They are thus not delivering enough information across time to form a basis for constructing quantifications of regional business cycles as monthly growth rates.

In addition, as it is the explicit aim of the Global Barometers to indicate the global development, referring to the same global target series for all regional aggregates ensures that our approach, whilst effectively correcting the variables’ weights by regions, directly focuses on global economic development. Hence, we adopt the following procedure to address the regions issue: We assign all indicator variables to one of the regions Europe, Asia and Pacific and Africa, and the Western Hemisphere. Currently, only these three regions are referred to for the breakdown, as further disaggregation would lead to regional aggregates for which only a limited number of variables are available so that the validity of the related factors would likely suffer. On this basis, the following applies:

- The variable selection is performed using the global reference series.
- A PLS factor is extracted for each region separately. The global reference series is used as response variable for all regions.
- The regional PLS factors are aggregated with the latest annual GDP shares as weights. (We impute the missing one or two years of the weight variables by forecasting the regional annual GDP growth rates with an AR(2) model.)

Tables 4 and 5 summarize descriptive statistics for the coincident as well as the leading Barometer, computed from the 2018m12 vintage (where “m” denotes the month). They show the percentages of available variables for every region, the percentages of selected variables and the shares of the sums of their absolute partial least squares’ weights. The last columns show the regions’ shares in PPP adjusted global GDP to indicate over- or underrepresentation, which is addressed by using these shares as weights in the aggregations.

**Table 4:** Regional shares for coincident Barometer 2018m12

Region	Available variables	Selected variables	Abs. PLS weights	World GDP
Europe	59%	63%	64%	21%
Asia and Pacific and Africa	21%	16%	16%	55%
Western Hemisphere	19%	21%	20%	24%

**Table 5:** Regional shares for leading Barometer 2018m12

Region	Available variables	Selected variables	Abs. PLS weights	World GDP
Europe	59%	59%	58%	21%
Asia and Pacific and Africa	21%	20%	23%	55%
Western Hemisphere	19%	21%	19%	24%

### Construction in pseudo real-time

To assess how well the two Global Economic Barometers would have indicated the world business cycle in real-time, we compute both Barometers in “pseudo” real-time for every month from 2002m1 to 2018m12, coinciding with the end of the last quarterly IMF world GDP data point. The attribute “pseudo” refers to the fact that we have access to real-time vintages neither for the indicator variables nor for global GDP over the relevant period and therefore have to resort to pseudo real-time vintages using the 2018m12 vintage.^27^ For the pseudo real-time vintages, it is important to consider the historical publication lags of the variables. A vintage in period $t$ of a variable with publication lag $l$ will exhibit observations up to period $t-l$, assuming that the publication lag of the variable is constant over time. For the start date of the pseudo real-time period, we must consider that our in-sample period for the variable selection is 10 years and only variables with complete observations across these 10 years can be processed. The number of available variables therefore decreases the further we go back in time.

**Fig. 3:**
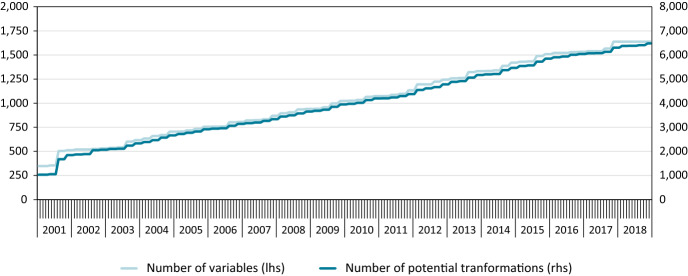
Number of variables with valid observations throughout the 10-years sample period across Barometer vintages

Figure 3 plots the number of available variables for every month starting in 2001m1 and ending in 2018m12. In early 2001, we would have been able to work with only about 1000 transformations. This almost doubles when moving to 2002. To avoid this sudden increase in available variables, we decided to set the start of the pseudo real-time period to 2002m1.

## The resulting Global Economic Barometers

**Fig. 4:**
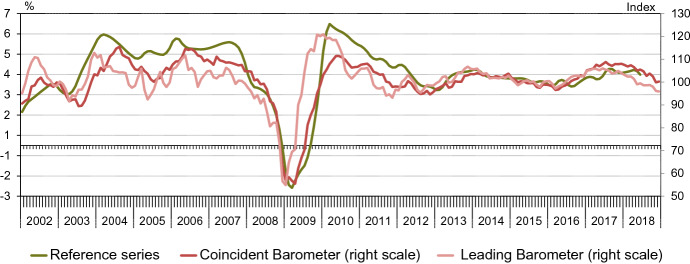
Global Economic Barometers in pseudo real-time and reference series

Figure 4 shows the resulting coincident and leading Global Barometers together with our monthly reference series. All observations for the Barometers are pseudo real-time and out-of-sample.

Several observations can be made. First, the leading indicator indeed appears to be leading both the coincident indicator and the reference series, in particular in signalling the major turning points of the reference series. Second, refraining from low-pass filtering promotes the endpoint stability of the Barometers, as desired. Yet, the leading Barometer appears more volatile than the coincident, especially in the earlier years. A reason for the higher volatility in the earlier part is probably the relatively limited number of variables from which the procedure can select those with the desired leads.

**Fig. 5:**
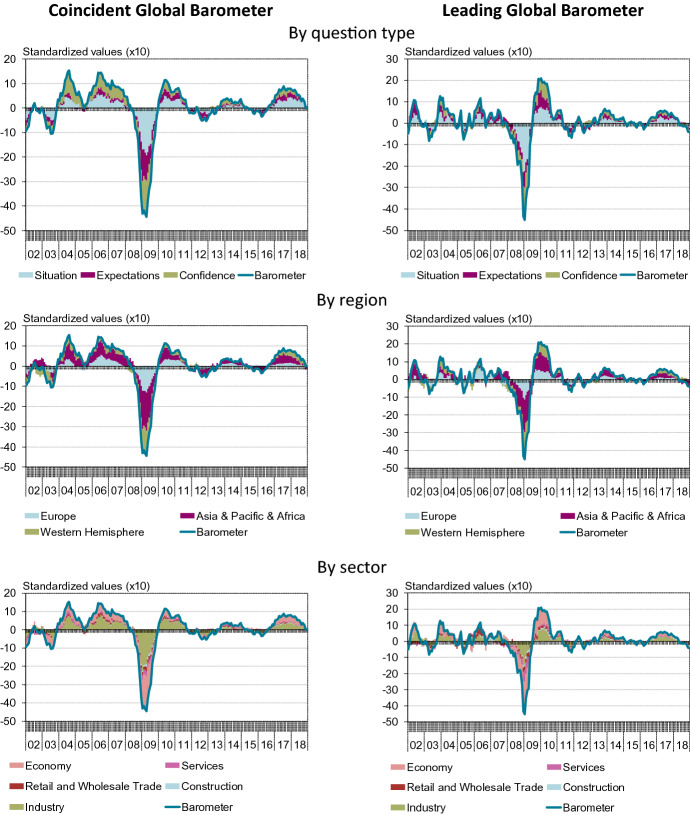
Contributions to the Barometers along different dimensions

To analyse what is driving the Barometers, we can classify the different variables into different groups. Here, we distinguish the selected variables by indicator/question types (present situation, expectations, or confidence), by regions (Western Hemisphere, Asia and Pacific and Africa, and Europe), or by sectors (Industry, Construction, Retail and Wholesale Trade, Services, and general Economy). These classification dimensions are shown in the three panels of Fig. 5.

Some remarks are in order. First, contrary to the expectation that the coincident indicator will be largely driven by assessments of the current situation and the leading indicator by more forward-looking questions, across the years under consideration here, this distinction is not readily observable (upper panel). Second, the panel in the middle shows that that the three broader regions that we distinguish all contribute noticeably to the Barometers. Third, the lower panel of the figure makes clear that the information from the manufacturing industries as well as from the economy as a whole are the main drivers of the Barometer from this sectoral perspective.

### Revision properties

The Global Barometers are updated monthly. In line with our objective to design a system of indicators that adjusts to the ever-changing economic circumstances, the entire selection and computation procedure is re-run every month, reflecting the frequency of most of our tendency survey data. For the reference series, however, updates are performed on a quarterly basis, when new quarterly observations for world GDP output growth are made available.^28^ This means that we should expect some corrections to all Barometer values every month. Every third month, however, the corrections should be somewhat more pronounced. For the average revisions, we should expect a mean of zero, unless there are systematic biases. This comes very close to what we observe; the averages^29^ for the revisions amount to − 0.009 for the coincident and to − 0.013 for the leading Global Barometer. Given that that mean scores of the Barometers are scaled to 100 with a standard deviation of 10, these deviations from zero are far from statistically significant and, most importantly, economically negligible. The empirical pattern also confirms that every third month revisions turn out to be more pronounced. Another observation is that the absolute revisions are higher on average for the leading Global Barometer than for the coincident one. For the coincident Barometer, the average absolute revision is 0.55 for the second months of the quarter and 0.05 for the remaining eight months of a year. For the leading Barometer, these are 0.87 and 0.11, respectively. Part of these differences could be due to the fact that a longer lead in the cross-correlations increases the proportion of marginally significant variables, so that changes in the reference period, which are likely to affect the selection of marginally significant variables, lead to less stability over time.

### Robustness tests

We now summarise the outcomes of a number of robustness tests to check whether our methodology is indeed preferable to some straightforward alternatives.^30^ For the first test, we omit the variable selection procedure and extract the PLS factor from all variables in the pool. For the second test, we do not aggregate the Barometers from regional PLS factors but compute them directly from the entire sets of variables that pass the coincident and leading selection procedures, respectively. For the third test, we extract principal components instead of PLS factors.

We assess how well our baseline Barometers are doing vis-à-vis the above-mentioned alternatives by evaluating how well they forecast the reference series over the pseudo real-time period from 2002m1 to 2018m12. To this end, we construct the Barometer vintages for all versions in pseudo real-time and use them to make a straightforward forecast of the reference series for various forecast horizons. The in-sample period is set to 10 years, ending with the last month of the reference series vintage. The forecasts are evaluated by computing the correlations and the root mean square errors (RMSE) of the forecasts with the 2018 vintage of the reference series. The horizon at which the correlation peaks is taken as the lead of a Barometer. The ideal Barometer should not only exhibit a high correlation and low RMSE at the desired lead, but also exhibit a peak in the correlation at the desired lead. Consequently, the coincident Barometer should have its highest correlation with a lead/lag (horizon) of zero and the leading Barometer with a lead of six months. In combination, we will refer to these desirable properties as the *quality* of the Barometers. In addition to the RMSE, we run one-sided Diebold-Mariano (DM) tests (Diebold & Mariano, 1995) with the alternative hypothesis that the baseline Barometer has a lower forecast error.

Recall that we expect to improve the quality of the Barometers by discarding variables with low correlations with the reference series or undesirable lead/lag properties, as our baseline Barometers rely on the “targeted predictors” approach. Comparing the RMSE for the baseline coincident Barometer and *versions without variable selection*, the baseline version has a higher correlation and a lower RMSE at a horizon of zero and the difference is significant at the 10%-level. For the leading Barometer, the baseline version peaks at a horizon of 4 months, closer to the target lead of 6 months than the version without variable selection, which peaks at a horizon of 3 months. The baseline version also has a lower RMSE at a horizon of 6 months, and the difference is significant at the 10%-level. The variable selection step thus improves the lead of the leading Barometer and the predictive accuracy of both Barometers.

Above, we identified regional overrepresentation in the variable pool as a potential problem and therefore chose to compute the baseline Barometers from three individually extracted regional PLS factors, targeted to global GDP, and aggregated with the corresponding regional GDP shares. Comparing the baseline coincident Barometer and *versions without regional aggregation*, the baseline version has a higher correlation and a lower RMSE at a horizon of zero and the difference is significant at the 10%-level. For the leading Barometer, the baseline version peaks at a horizon of 4 months, whilst the version without regional aggregation peaks at a horizon of 5 months, which is closer to the desired lead of 6 months. However, the baseline’s correlation at a horizon of 6 months is much higher and the RMSE is significantly lower at the 10%-level. In conclusion, the regional aggregation leads to better predictions at the expense of a slight loss of lead of the leading Barometer.

Furthermore, although we have stated good reasons for resorting to PLS in the aggregation procedure, PLS is still a rare choice in the macroeconomic forecasting literature. The most prominent approach for constructing composite indices is the approximate factor model introduced by Stock and Watson (2002a, 2002b), who show that factors can be consistently estimated using principal components if $N$ is sufficiently large. In our case, we will compare our baseline Barometers to alternative Barometers estimated with principal components. Comparing the baseline coincident Barometer and *versions using principal components*, the baseline version has a slightly higher correlation and a lower RMSE at a horizon of zero; the difference is significant at the 10%-level. For the leading Barometer, at a horizon of 6 months, the baseline version also has a slightly higher correlation and a lower RMSE, although the difference is not significant at conventional levels. Overall, during the period under consideration, our PLS baseline versions appear preferable to the PC alternatives.

While the differences between our Barometers and the alternative versions are not particularly pronounced throughout, they consistently point into directions that support our choices.

## Comparing the new Global Economic Barometers with other composite leading indicators for the world economy

This section compares the Global Barometers with some prominent alternatives. Section 2 concluded that comparable indicators for the world economy comprise those of the OECD, the Conference Board, ifo and Markit Economics.

The composite leading indicators of both the OECD and The Conference Board differ regarding the underlying business cycle concept. The OECD indicator focuses on growth cycles. To measure this, de-trended GDP is used.^31^ The trend is removed by applying the Hodrick-Prescott filter (Hodrick & Prescott, 1997). On the other hand, The Conference Board indicators target economic expansions and contractions. In this sense, the indicators try to signal the classical business cycle that looks at fluctuations in the level of economic activity. However, the Conference Board Leading indicator can be “de-trended”. As this indicator is calculated with symmetric growth rates, we can calculate a stationary indicator with the inverse function.^32^ Other candidates for comparison are the ifo World Economic Climate Indicator and the Markit PMI indicator. Both are based on surveys amongst experts and firms across countries, relying on a very limited number of survey items. They do not target a specific economic magnitude, but they appear to be taken as indicators for the growth of the world economy.

All aforementioned composite indicators are based on comparatively small sets of “hand-selected” variables.^33^ Whilst such parsimony is not a necessarily detrimental to the usefulness of the indicators, it implies that economic experts must continuously monitor the performance of the variables and adjust the selection whenever this becomes necessary. In contrast, the impact of individual expertise is limited for the maintenance of the Global Barometers. Their underlying variable selections and the ensuing computations are transparent and non-subjective. We have well-defined rules, which are carried out every month. Finally, yet importantly, the Global Barometers are freely available, but access to the Markit PMI indicator and the Conference Board indicator is given only to paying customers, so that we unfortunately have to exclude them from the following calculations.

**Fig. 6:**
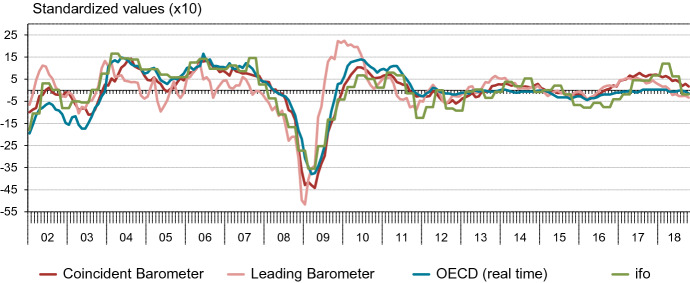
Selected indicators for the world business cycle

Accordingly, we compare the Global Barometers with the OECD and the ifo World Economic Climate indicator. Figure 6 shows the four indicators, covering the same period as the last vintage of our last IMF reference series, and transformed to the same means and variances to ease comparison.

In relation to the OECD indicator, the ifo indicator is coincident according to the cross-correlation function (*r* = 0.85). The same holds for our coincident Barometer (*r* = 0.90). The leading Barometer, on the other hand, has its maximum cross-correlation at a lead of three months (*r* = 0.77) before the OECD indicator. This chronology supports the claim that we have indeed constructed a set of two indicators, where one is leading in terms of the other. In practice, publication lags play a key role, as they overstate leads in real-time. The monthly OECD leading indicator is released about two months after the reference period. The quarterly ifo World Economic Climate indicator is published in the first half of the second month of the reference period.

In commonly used forecasting horse-race exercises, lags of the target series are used for the forecast. To address this, we first choose a common target series, namely quarterly global real year-on-year GDP growth, published by the IMF. The GDP vintage that ends at the end of 2018 is used in a pseudo real-time setup. To simulate the real-time situation, we must also consider the publication lag of the reference series. According to the IMF, there is no fixed timeframe for the release of this time series. Bases on experience from the past, data for the first quarter are often published in July, for the second quarter in September or October, for the third quarter in January and for the fourth quarter in April. This means that a reasonable assumption for the real-time exercise is that the data for a particular quarter are published after the end of the following quarter. Accordingly, we cannot include an AR(1) term in AR model for GDP; lags of GDP can start with *t* − 2. Regarding the indicators, we use the following setup:

- OECD composite leading indicator: Real-time vintages are publicly available on the OECD website. Vintages are available at a monthly frequency since January 2001.
- ifo World Economic Climate: Real-time vintages are not available; the analysis must hence be conducted in pseudo real-time. However, this should not cause major distortions, as the indicator is usually not revised.
- Global Economic Barometers: Since real-time vintages of the underlying variables are not available, we refer to pseudo real-time vintages starting in January 2002.

The setting of the simulated real-time experiment is as follows. Starting from April 2002, we begin by forecasting annual GDP growth for the third quarter of 2002, using time series dating back to 1992. This allows us to use an estimation period of 10 years. Over time, we calculate rolling out-of-sample forecasts so that we always have the same length of the underlying time series. As we are not increasing the sample size, we can apply the Giacomini White approach to test nested hypotheses regarding forecasting performance. We simulate the real-time forecasting situation, starting in the first month of the quarter before the reference quarter and ending with the situation in the last month of the reference quarter. Since the OECD indicator and the Global Barometers are monthly indicators and the target series of GDP is at quarterly frequency, we have to deal with mixed frequencies. The unrestricted MIDAS approach (U-MIDAS) is applied here. We allow for up to three lags in the GDP autoregressive part and up to nine lags for the monthly indicator variables, excluding publication lags. For each forecast, a specific model is selected by the Bayesian Information Criterion (BIC). In general, the model for the monthly indicators can be written as follows:

$$y_{t} - \alpha_{1} y_{t - 1} - \ldots - \alpha_{p} y_{t - p} = \mathop \sum \limits_{i = 0}^{k} \mathop \sum \limits_{j = 0}^{{l_{i} }} \beta_{j}^{\left( i \right)} x_{{tm_{i} - j}}^{\left( i \right)} + \varepsilon_{t} ,$$

where $y_{t}$ denotes the low frequency series and ${x}_{t}$ the high frequency series. For quarterly $y$ and monthly $x$, $m=3$. In the present case, $p=3$ and ${l}_{i}=9$ with $i=1$. For the monthly indicators, lags of $x$ are deleted according to the publication lags. For the quarterly ifo World Economic Climate an ordinary Autoregressive Distributed Lag Model is estimated. Table 6 shows the Root Mean Squared Errors (RMSE) of the rolling out-of-sample forecasts for the different indicators.^34^ The inclusion of indicators in the AR model always leads to a reduction in the RMSE, and mostly in a statistically significant way.

**Table 6:**
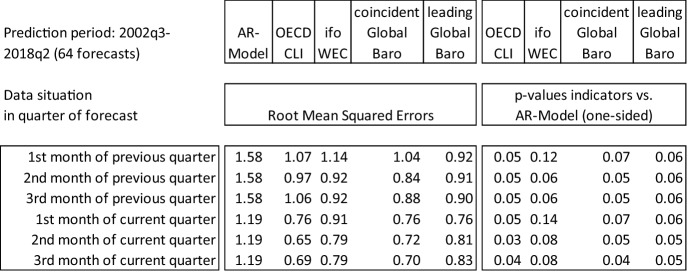
Comparing forecasting performance of different global indicators

As the table shows, the coincident Barometer has its strengths in the quarter before the reference quarter. In the second and third months of the previous quarter, the RMSEs are relatively small compared to the OECD indicator. The leading Barometer performs relatively well in the months before the reference quarter compared to the OECD indicator. Especially in the first month of the previous quarter, the leading Barometer provides additional information. The right part of the table shows the p-values of Giacomini White tests. Models containing the OECD indicator always perform significantly better than the AR approach, which is taken as the benchmark. In addition, the coincident and leading Barometers both outperform the AR approach, with p-values ranging from 0.04 to 0.07. The situation for the ifo indicator is less clear. It is never significant at the 5%-level, with p-values ranging from 0.06 to 0.14. It performs best in months two and three of the previous quarter. Tests to discriminate between the indicators, rather than between the indicators and the AR-benchmark, show that there are in general no significant differences between the different indicators.^35^

Taken together, these comparisons confirm that the Global Barometers are a useful addition to the alternatives that are presently released on a regular basis and freely available to the public.

## The Global Barometers during the Covid-19 pandemic

The Covid-19 pandemic and its drastic impact on economies around the world pose a challenge to economic forecasting unprecedented in the absence of wars, revolutions, or similar shocking disruptions. All non-esoteric forecasting extends regularities from the past into the future. Realising the break-down of most typical causing factors and sequences of economic recessions, busts and recoveries, economic forecasters were quick to look for alternatives to the business and usual and developed a host of innovative indicators and models.^36^ In particular, indicators that are based on current information and do not work with autoregressive terms tend to react relatively quickly to changing circumstances. Our Global Barometers belong to this group.

Although the monthly releases of the Global Barometers started in January 2020, just before the global economy would slide into one of the most dramatic slumps ever, their methodology, data basis and calibration were developed in the years before. Yet, while we were well aware of the Great Recession in the wake of the 2007 US financial crises and devoted considerable effort to make the Barometers robust against such events, we still relied on established economic reasoning. In particular, the data generating processes underlying the Barometers are the respondents’ replies to economic surveys, which link past experience to the current set of information. Given the novelty of the pandemic with no relation to living memory, this presents a serious challenge. Did the Global Barometers stand this test? As we will show, the Global Barometers were at least quick to signal the recession in spring 2020, the rebound in summer, the strong recovery in spring 2021 and the following slow-down in summer and autumn.

At the time of writing, the latest vintages of the Global Barometers are from October 2021. In February 2020, first reports appeared of Covid-19 spreading out of China and getting out of control. Subsequently, people started to take precautionary measures and restrictions were imposed worldwide to contain the spread of the virus. As a result, numerous economic activities were constrained or brought to a complete standstill. In economic terms, the pandemic is an exogenous shock of non-economic origin. Under typical cyclical circumstances, when recessions are generated within the economy and gradually spread through economic mechanisms that may at least be intuitively accessible to the respondents of the surveys, a lead of a few months in anticipating upcoming economic tendencies in the near future may be realistic. At the eve of the Pandemic Recession, however, the vast majority of economic observers grossly underestimated the exponential increase of infections that became known in March 2020; and they lacked the imagination to anticipate the drastic reduction of economic activity that followed suit. As a result, the forward-looking Barometer showed no lead. Though in this case a lead time of economic indicators could hardly be expected, we should at least demand that the Global Barometers, both the coincident and the leading one, indicated the crisis immediately after it happened. It is precisely in such situations that economic agents want information on the state of the economy as soon as possible.

**Fig. 7:**
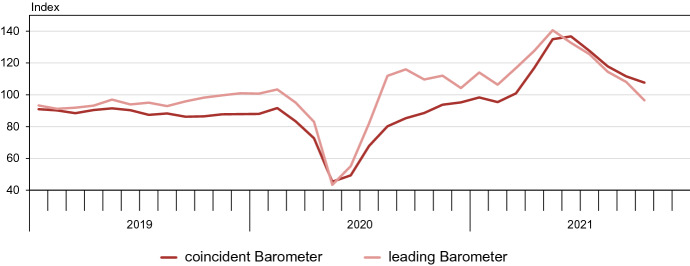
The Global Barometers and the 2020 pandemic (vintages of 2021m10)

As Fig. 7 demonstrates, the Global Barometers passed this test convincingly. After a last peak in February 2020, they embarked on a plunge to meet their troughs in May. The steep descents of about to 45 points for the coincident Barometer and close to 60 points for the leading one, corresponding to close to 4.5 and 6 standard deviations, respectively, are truly remarkably and unique in the Barometers’ time series, which go back to 1991. After the low point in May, the leading Barometer in particular rose significantly during late spring and summer and, with the August release, climbed above its long-term average. It thus pointed to a rapid rebound. The coincident Barometer tended to follow the direction of the leading Barometer but remained below its long-term average for the rest of 2020. The second wave of the pandemic, especially in the USA and Europe, with also rising Covid case numbers in some other countries, such as Brazil, may have caused the factual development to fall short of expectations. The leading Barometer tended to move sideways during autumn and early winter of 2020/21. According to the Global Barometers, the second wave of COVID did not lead to a renewed collapse of the economy as in spring 2020. From March 2021, the leading Barometer again climbed sharply. The vaccination campaigns were likely to have spurred this optimism. The coincident Barometer followed and also rose strongly. In the first estimates of the statistical offices for GDP in the second quarter, some large economies show a quite positive development. Quarter-on-quarter real GDP growth in the USA was annualised 6.5% (12.2% year-over-year), in the EU annualised 8% (13.2% year-over-year). In China, the dynamic was a bit slower because a stronger rebound had already taken place earlier. But even in China, according to initial estimates, there was a quarter-on-quarter increase in the second quarter of 2.9% (7.9% year-over-year).

The latest release of the Global Barometer reports (October 2021) shows that the leading indicator has fallen five times in a row after peaking in May. In October, it fell below its long-term average. This suggests that the strong expansion seen in spring will considerably lose momentum. The coincident Barometer started to fall again in July, confirming this tendency, but in October it nevertheless still above its long-term average. The picture at the current edge also shows that a joint look at the two Global Barometers can help to understand the dynamics in real time, as the information from the two indicators is complementary. A signal from the leading indicator can be confirmed by further signals from this indicator in the following months and additionally by the coincident indicator. If it is not confirmed, it may indicate uncertainty about the tendency.

## Summary and conclusions

The Global Economic Barometers are designed to provide the public with a set of two quantitative composite indicators for the world economy; one of them coincident, the other leading. The principle building blocks are the identification of theoretically valid variables with empirically established leads before the reference series, or synchronicity with it, and the subsequent aggregations of these variables. In particular, we compute the first partial least squares components from two pre-selected bundles of variables with the same reference series—an approximation of the annual growth rate of world GDP at monthly frequency—but with different lead-lag patterns. To enhance the robustness of the resulting Global Barometers, we impose structure in the form of regional weights.

The performance of the Barometers is illustrated by means of pseudo real-time simulations. The main conclusion is that the coincident Global Barometer indeed tracks the reference series well, and the leading Global Barometer comes close to the targeted lead of about six months, which is particularly visible at major turning points. As expected, the lead comes at a price, which manifests itself in a higher volatility of the leading as compared to the coincident indicator, so that the latter’s congruence with the references series is more pronounced.

We then compared the congruence and the leading properties of the two Barometers with the OECD composite leading indicator and the ifo World Economic Climate indicator, presently the only other indicators for the world business cycle that are publicly available, i.e. not hidden behind a paywall. We find that the coincident Barometer has its strengths in the quarter before the reference quarter and the leading Barometer performs relatively well in the months before the reference quarter.

We then summarised the results of three robustness tests by changing selected features of the Barometers: an alternative without variable pre-selection; an alternative without regional aggregations and an alternative aggregation by extracting the first principal component rather than the first PLS factor. We find that the alternative specifications do not improve the properties (evaluated as combinations of leads with respect to and correlations with the reference series) of neither the coincident, nor the leading Global Barometer.

Finally, we had a close look at the performance of the Global Barometers at the onset of the Covid-19 pandemic recession in March 2020, the quick recovery only months later and the following bumpy evolution up to October 2021. We find that despite the difficulty to foresee economic developments in such an extraordinary situation, the Global Barometers to a large degree retained their properties.

Given our real-time analyses, along with a first look at the immediate consequences for the world economy in the pandemic, we feel confident to conclude that the Global Barometers constitute useful additions to the small number of indicators for the global business cycle published so far.

## Footnotes

1 Confidence surveys are now conducted in more than 90 countries; for an overview and an assessment of the leading properties of the data they reflect, see Ha et al., 2019.

2 They are published online at monthly frequency since January 2020; see https://kof.ethz.ch/en/forecasts-and-indicators/indicators/kof-globalbaro.html.

3 See e.g. Gregory et al. (1997), Ambler et al. (2004), Graff (2006) and Layton et al. (2015).

4 See https://www.unido.org/resources/statistics/quarterly-report-manufacturing.

5 See https://www.cpb.nl/en/worldtrademonitor.

6 See https://data.oecd.org/industry/industrial-production.htm.

7 See https://hwwi-rohindex.de.

8 See https://www.balticexchange.com/.

9 See https://www.worldsteel.org/steel-by-topic/statistics.html.

10 See https://www.iata.org/en/publications/economics?cat=Air%20Freight%20Monthly%20Analysis&menu=Market%20Developments&cat=Air%20Freight%20Monthly%20Analysis&menu=Market%20Developments.

11 See http://www.cesifo-group.de/ifoHome/facts/Survey-Results/World-Economic-Survey.html.

12 See https://www.markiteconomics.com.

13 See https://www.conference-board.org/data.

14 See https://data.oecd.org/leadind/business-confidence-index-bci.htm#indicator-chart.

15 See https://data.oecd.org/leadind/consumer-confidence-index-cci.htm#indicator-chart.

16 See https://ec.europa.eu/info/business-economy-euro/indicators-statistics/economic-databases/business-and-consumer-surveys_en. The non-member economies are Brazil, China, India, Indonesia, Russian Federation and South Africa.

17 See https://data.oecd.org/leadind/composite-leading-indicator-cli.htm#indicator-chart.

18 See https://www.conference-board.org/data.

19 For the reference series underlying the monthly versions now published on https://kof.ethz.ch/en/forecasts-and-indicators/indicators/kof-globalbaro.html, KOF extends the IMF’s last published y-o-y world GDP growth rate vintage with its own estimates based on official national statistics. None of the authors is directly involved in this. As soon as the IMF resumes its publication, we will revert to that source again. (To the best of our knowledge, whilst annual world GDP data are available from various sources, the IMF is the only institution publishing world GDP data at quarterly frequency.).

20 The objective of the Denton additive method is a temporal distribution of the quarterly values to a monthly frequency in a way that keeps the difference between the estimated monthly series and an indicator series as constant as possible, subject to quarterly constraints. Since in the present case a constant is used instead of an indicator, the additive Denton method is similar to the first-difference approach of Boot et al. (1967). The procedure is not endpoint stable. To construct a value for period *t*, it also uses information in period *t* + 1 and beyond. For a discussion of various methods for temporal disaggregation, see Chen (2007).

21 The current variable catalogue, along with the most important meta-data, can be found at https://kof.ethz.ch/en/forecasts-and-indicators/indicators/kof-globalbaro.html.

22 E.g. Abberger et al. (2018) for Switzerland; Duarte and Süssmuth (2018) for Spain.

23 For an overview of variable selection methods for partial least squares, see Mehmood et al. (2012).

24 This is the result of judgement based on economic reasoning. In some instances, the expectations are ambiguous, and we leave the outcome undetermined.

25 For technical reasons related to the pandemic, we are keeping the window for the reference series fixed since May 2020, covering the period from January 2010 to December 2019. As noted above (see footnote 19), until the IMF resumes publishing world GDP at a quarterly frequency, the quarterly data points that are used to impute the monthly reference series are KOF estimates.

26 For a related problem that is likewise addressed via disaggregation, see Graff (2010).

27 We tried to collect real-time vintages of the IMF’s y-o-y world GDP growth rate from different sources, however with only partial success. The gaps between the retrieved vintages make it difficult to draw strong conclusions based on such data. We therefore stick to the 2018m12 vintage of the IMF’s y-o-y world GDP growth rate throughout the analyses reported in this paper.

28 As mentioned above (see footnotes 19 and 25), as long as the IMF does not resumes publishing quarterly world GDP data, the quarterly data points that we use to impute the monthly reference series are KOF estimates.

29 Notice that in contrast to the performance evaluations of the Global Barometers, which require vintages of the reference series and hence cannot be conducted for observations later than 2018m12, this exercise can resort to all data points of the Barometers.

30 Readers interested in the technical details of the robustness tests are referred to Abberger et al. (2020). The R code and data to reproduce these results can be found at https://kof.ethz.ch/en/forecasts-and-indicators/indicators/kof-globalbaro.html.

31 Before 2012, the OECD used a de-trended index of industrial production; see Fulop and Gyomai (2012).

32 The trend in the indicator *I*_*t*_ is removed by ${x}_{t}=200 ({I}_{t}/{I}_{t-1}-1)/({I}_{t}/{I}_{t-1}+1)$.

33 See OECD (2012) and The Conference Board (2001, pp. 1315) for the selection procedures.

34 The R code and data to reproduce this table can be found at https://kof.ethz.ch/en/forecasts-and-indicators/indicators/kof-globalbaro.html.

35 The results of these tests are available upon request.

36 For an overview, see Haldane and Chowla (2021).
